# Metabolic systems analysis of LPS induced endothelial dysfunction applied to sepsis patient stratification

**DOI:** 10.1038/s41598-018-25015-5

**Published:** 2018-05-01

**Authors:** Sarah McGarrity, Ósk Anuforo, Haraldur Halldórsson, Andreas Bergmann, Skarphéðinn Halldórsson, Sirus Palsson, Hanne H. Henriksen, Pär Ingemar Johansson, Óttar Rolfsson

**Affiliations:** 1 Center for Systems Biology, University of Iceland, Sturlugata 8, Reykjavik, Iceland; 2 Medical Department, University of Iceland, Sturlugata 8, Reykjavik, Iceland; 3 Landspitali, Læknagarður, Hringbraut, Reykjavik, Iceland; 4 Rigshospitalet, Blegdamsvej 9, 2100 Kobenhavn O, Denmark

## Abstract

Endothelial dysfunction contributes to sepsis outcome. Metabolic phenotypes associated with endothelial dysfunction are not well characterised in part due to difficulties in assessing endothelial metabolism *in situ*. Here, we describe the construction of iEC2812, a genome scale metabolic reconstruction of endothelial cells and its application to describe metabolic changes that occur following endothelial dysfunction. Metabolic gene expression analysis of three endothelial subtypes using iEC2812 suggested their similar metabolism in culture. To mimic endothelial dysfunction, an *in vitro* sepsis endothelial cell culture model was established and the metabotypes associated with increased endothelial permeability and glycocalyx loss after inflammatory stimuli were quantitatively defined through metabolomics. These data and transcriptomic data were then used to parametrize iEC2812 and investigate the metabotypes of endothelial dysfunction. Glycan production and increased fatty acid metabolism accompany increased glycocalyx shedding and endothelial permeability after inflammatory stimulation. iEC2812 was then used to analyse sepsis patient plasma metabolome profiles and predict changes to endothelial derived biomarkers. These analyses revealed increased changes in glycan metabolism in sepsis non-survivors corresponding to metabolism of endothelial dysfunction in culture. The results show concordance between endothelial health and sepsis survival in particular between endothelial cell metabolism and the plasma metabolome in patients with sepsis.

## Introduction

Sepsis is a life-threatening systemic inflammatory condition caused by the body’s response to infection, leading to fever, increased heart and breathing rates and septic shock is the subset of these patients with hypotension and altered serum lactate^1–3^. The estimated annual cost of sepsis hospitalization in the US is around $15 billion^4^, with mortality rates around 30%^5^. An NIH panel recently redefined sepsis as “severe endothelial dysfunction syndrome in response to intravascular and extravascular infection”^6,7^. All blood vessels of the body are lined with a single layer of endothelial cells. These cells are the contact-point between blood and the body and as such they play a pivotal role in sepsis. Endothelial dysfunction refers to the inability of the endothelium to produce appropriate concentrations of vasorelaxants, particularly nitric oxide (NO), to properly control blood pressure^7^. Furthermore, endothelial injury due to sepsis leads to oedema and plays a role in dysregulation of blood clotting^8–10^. Patient response to treatment is somewhat endothelium dependent and highly variable^11^. Altered metabolism has been described in sepsis patients^12^. Metabolism in sepsis is reflected in both treatment guidelines and broader studies identifying metabolic fingerprints for sepsis outcome prediction^13–16^. Plasma metabolites in sepsis patients are similar to those of healthy lipopolysaccharide (LPS)-treated volunteers^15,17,18^, suggesting that LPS provides a useful proxy for sepsis studies. Interferon γ (IFNγ) has been shown to potentiate LPS stimulation in cultured human umbilical vein endothelial cells (HUVECs)^19^.

Studies have implied a role for endothelial metabolism in many diseases, specifically glycolysis and glycosylation of surface proteins^20,21^. Endothelial permeability is dependent on fatty acid oxidation^22^ and arachidonic acid, ceramide, tryptophan, and arginine metabolism have all been shown to be important in sepsis progression^23–28^. Addressing endothelial metabolic changes may present novel avenues for specific treatment of sepsis. However, the full functional relevance of endothelial metabolic changes in sepsis are not fully understood. This knowledge gap is partly due to the difficulty of sampling directly from the endothelium *in vivo* and therefore of separating endothelial metabolism from that of other cell types^29^. To address this problem *in vitro* and *in silico* techniques are needed to analyse endothelial metabolism.

Genome scale metabolic models (GEMs) offer a framework for understanding metabolism through integrated analysis of gene expression, protein abundance and metabolic flux^30^. GEMs have previously been used to analyse the metabolism of cells *in silico* and applied to diverse problems, including biomarker identification^31^ and patient stratification of treatment^32,33^. A proteomics based GEM has recently been built for the endothelium and identified a role for carnitine palmitoyl transferase in endothelial permeability^22^. These developments present an intriguing possibility for the treatment of sepsis, given the variable patient response to treatment^11,29^. The quality of GEMs is dependent on manual curation by literature review and the coverage of context specific data^34^.

Here we describe the construction of a cell specific endothelial metabolic model, iEC2812, and its application to study septic endothelial dysfunction. We built and manually curated GEMs of three common endothelial subtypes and compared their output. We then focused our study on metabolic changes in cultured HUVECs stimulated with combinations of LPS and IFNγ to parametrize the model to endothelial dysfunction. Finally, we utilized iEC2812 as a framework to analyse publically available plasma metabolomics data from sepsis patients^35^, allowing endothelial metabolism in these patients to be inferred on the basis of a metabolic network reconstruction. We demonstrate that iEC2812 bridges a gap between *in vitro* and clinical research and represents a platform for the future systems analysis of endothelial metabolism.

## Results

### HUVECs, HMVECs and HPAECs have similar metabolism within iEC2812

**Figure 1:**
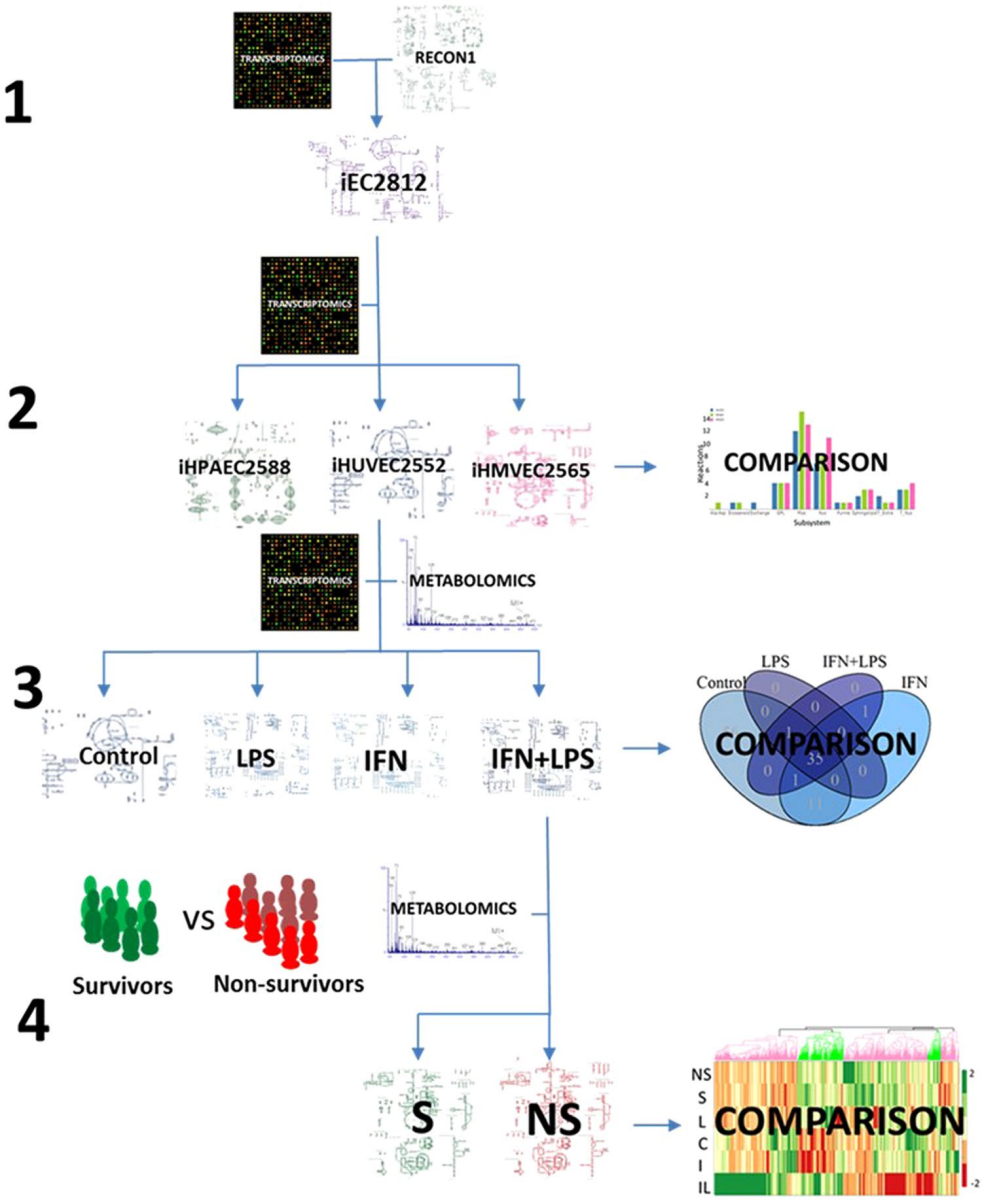
Schematic of the model building process illustrating sources of data and model outputs. The area indicated by label (**1**) shows the combination of RECON1 human genome scale metabolic reconstruction and transcriptomic data to form iEC2812. This is then combined, shown in the area labelled (**2**), with further transcriptomic data to form iHUVEC2552, iHPAEC2588 and iHMVEC2565. These models are compared to produce part of the results of this paper. More transcriptomic data is combined with iHUVEC2552 and metabolic data from cultured cells to produce models of cultured HUVECs treated with LPS and IFNγ to form four models shown in the section labelled (**3**), both the metabolic data used and the comparison of these four models form part of the results of this paper. The section labelled (**4**) shows the model of HUEVCs treated with both LPS and IFNγ being combined with metabolic data from the literature to model the endothelial metabolic differences between sepsis survivors and non-survivors, the comparison of these models forms the final part of the results section in this paper.

**Figure 2:**
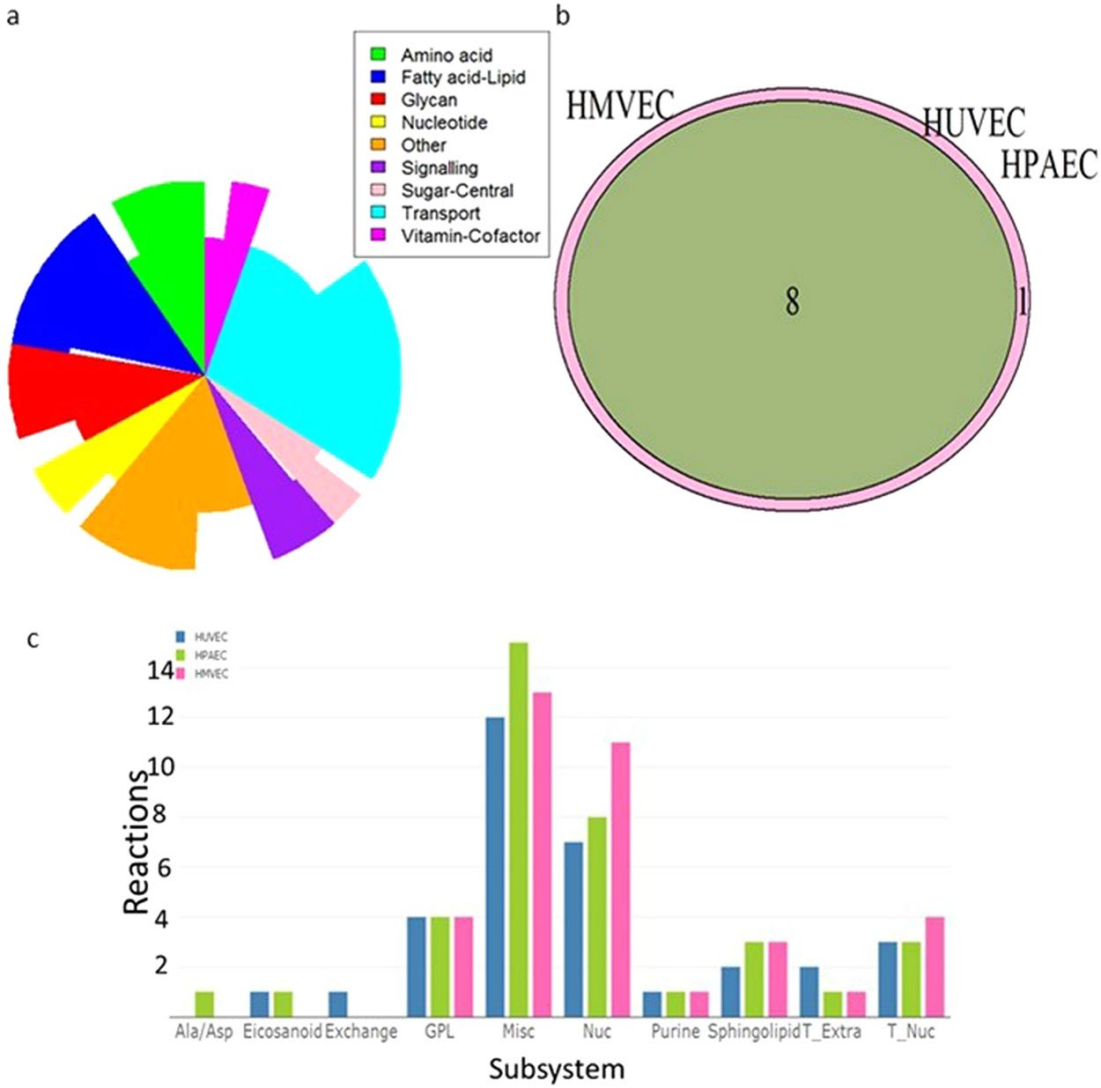
The structure, components and differences in metabolism of iEC2812 and comparison of the endothelial subtype genome scale metabolic models iHUVEC2552, iHPAEC2588 and iHMVEC2565. (**a**) The distribution of reactions in RECON1 (inner circle) and iEC2812 (outer circle) divided into metabolic super subsystems. (**b**) The majority of essential genes are shared between each sub-type model, iHUVEC2552 and iHPAEC2588 overlap totally and iHMVEC2565 has one unique essential reaction. (**c**) The classifications of essential reactions by subsystem*. Alanine/aspartatane, eicosanoids, exchange, glycerophosphlipid, miscellaneous, nucleotide, purine, sphingolipid, extracellular transport and nuclear transport*.

We built a metabolic reconstruction of endothelial metabolism called iEC2812 based on RECON1 (Fig. 1). Endothelial specific functions and relevant gene-reaction connections were added, spread across different areas of metabolism (Fig. 2a and Supplement vii). iEC2812 contains 2,023 genes and 2,812 reactions involving a total of 1,979 metabolites (Table 1). iEC2812 and its construction is described in detail in Materials and Methods and Supplements.

**Table 1:** The model components; reactions, total and those without a linked gene, metabolites, genes (total and inactive) of RECON1^42^, iEC2812 and endothelial specific subtype genome scale metabolic models and the genes in the data set linked to each model.

Model	Reactions	Metabolites	Genes (Active)	Metabolic Genes in dataset^54^	Unlinked Reactions
RECON1^42^	3,743	2,766	1,905 (983)	NA	1,436
iEC2812	2,812	1,979	2,023 (1,390)	1,003	851
iHUVEC2552	2,552	1,878	2,023 (1,411)	905	738
iHPAEC2588	2,588	1,915	2,023 (1,411)	870	742
iHMVEC2565	2,565	1,909	2,023 (1,384)	872	763

To examine endothelial metabolic diversity, we built and compared metabolic reconstructions of human microvascular endothelial cells (HMVEC- iHMVEC2565), human pulmonary artery endothelial cells (HPAEC- iHPAEC2588) and HUVECs (iHUVEC2552) (Supplements i and viii). All of the endothelial sub-type models contain pathways secreting NO, methoxy-tryptophan, L- kynurenine, sphingosine-1-phosphate, prostaglandin D2 and prostaglandin E2 and gamma amino butyric acid (GABA), all biomarkers of endothelial injury^27,36–40^. All of the models were capable of synthesising nucleotides from fatty acids which has been confirmed in endothelial cells^41^. The endothelial sub-type models were investigated by comparing essential genes and reactions using the RECON1 biomass function as the objective^42,43^. The majority of essential genes were found within nucleotide or complex lipid metabolism and were shared by all models (Supplement ii and Fig. 2b). Essential reactions unique to one sub-type model were largely in nucleotide metabolism (Fig. 2c).

In summary, the endothelial reconstruction iEC2812 and the three sub-type reconstructions iHUVEC2552, iHPAEC2588 and iHMVEC2565 contain relevant reactions and are largely similar in both metabolic component coverage and function.

### Functional and metabolomics analysis of LPS and IFNγ stimulation of HUVECs

LPS stimulation is a frequently used experimental system to mimic the effects of bacterial infection *in vitro* and *in vivo*^18,44,45^. To verify LPS function, we measured endothelial permeability and performed immunofluorescent imaging of total glycocalyx and the clinically relevant component heparan sulphate proteoglycan 2 in treated and untreated endothelial cells.

**Figure 3:**
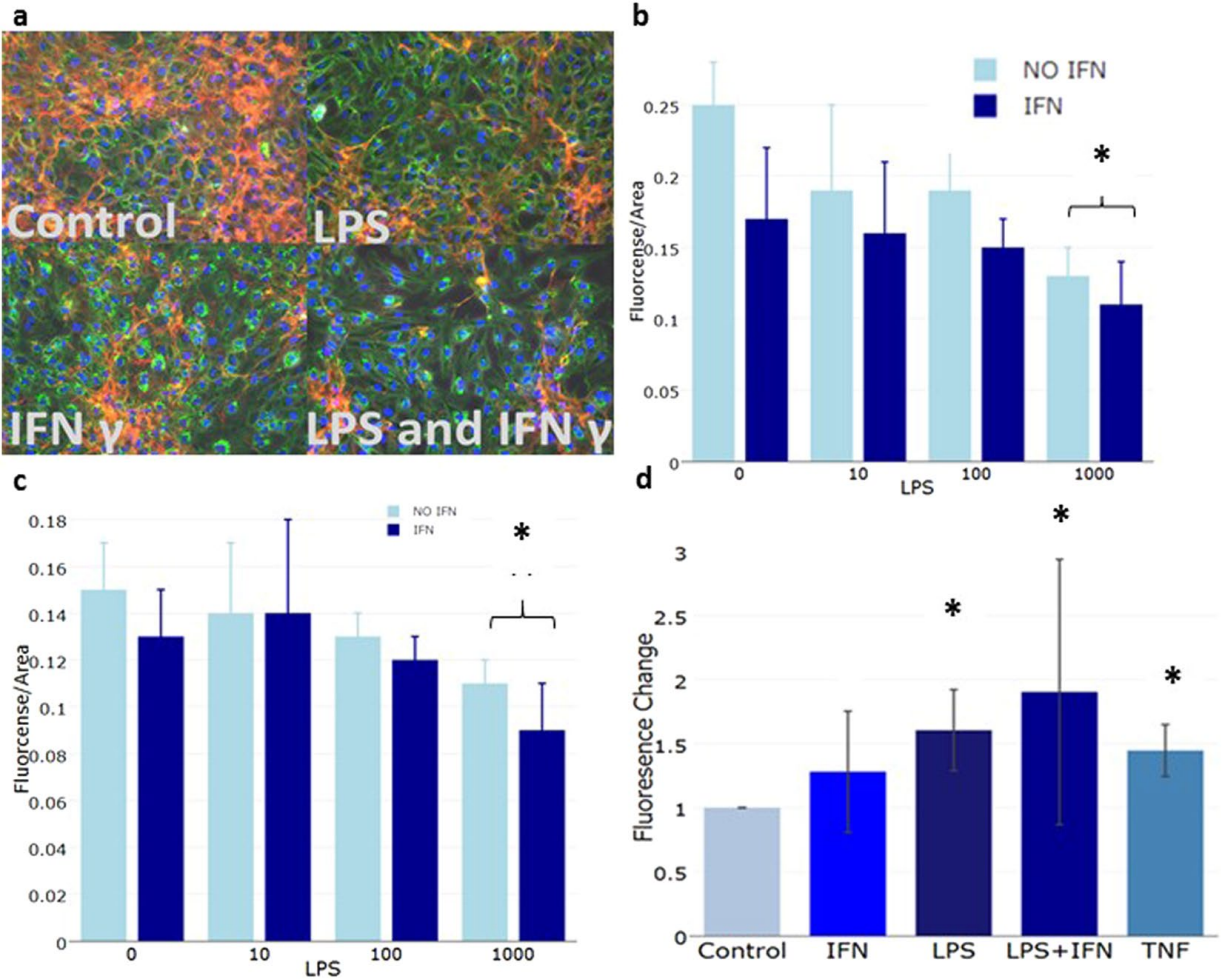
LPS and IFNγ treatment of HUVECs result in glycocalyx loss, increased permeability and changes in cellular metabolism. (**a**) Fluorescent images of HUVECs control or treated with 50 µg/mL IFNγ, 1000 ng/mL LPS for 24 h or a combination of the two (LPS/IFNγ) stained with DAPI (nucleus, blue), wheat germ agglutinin (negatively charged glycocalyx components, green) and heparan sulfate proteoglycan 2 antibody (red). (**b**) Normalized change in heparan sulfate signal by LPS concentration and IFNγ. Full statistics Supplement ixb. (**c**) Normalized change in wheat germ agglutinin signal by LPS concentration and IFNγ. Full statistics in Supplement ixa. (**d**) Normalized change in fluorescence, indicating permeability, in a transwell assay, over 45 minutes after treatment with IFNγ, LPS and tumour necrosis factor α (TNF α). (* significant at p < 0.05).

Total glycocalyx and heparan sulphate proteoglycan 2 staining decreased with either LPS or IFNγ treatment and to a greater extent when combined (statistical analysis in Supplement ix). HUVECs lost 27% (t-test, n = 5, p = 0.001) of their total glycocalyx in response to LPS stimulation alone, a non-significant decrease of 13% occurs (t-test, n = 5, p = 0.163) in response to IFNγ and up to 40% (t-test, n = 5, p = 0.001) in response to combined stimulation. Much of this change is due to loss of heparan sulphate proteoglycan 2 (Fig. 3a–c). A decrease of 48% (t-test, n = 5, p < 0.001) of the heparan sulphate proteoglycan 2 was seen in LPS treated cells. This further decreased to 56% (t-test, n = 5, p < 0.001) combined with IFNγ whilst IFNγ alone caused a 32% loss (t-test, n = 5, p = 0.023) (Fig. 3a–c and Supplement ix).

An increase in endothelial permeability accompanied the loss of the glycocalyx upon treatment (Fig. 3d). Endothelial permeability was increased by 60% with LPS treatment (n = 3, t-test, p = 0.008). A smaller, non-significant, increase was seen in IFNγ treated cells (t-test, n = 3, p = 0.058). The largest increase, 90%, was seen in cells treated with both LPS and IFNγ (t-test, n = 3, p = 0.043), consistent with the immunofluorescent staining. As reference, cells were treated with tumour necrosis factor α, a known inducer of endothelial permeability.

**Figure 4:**
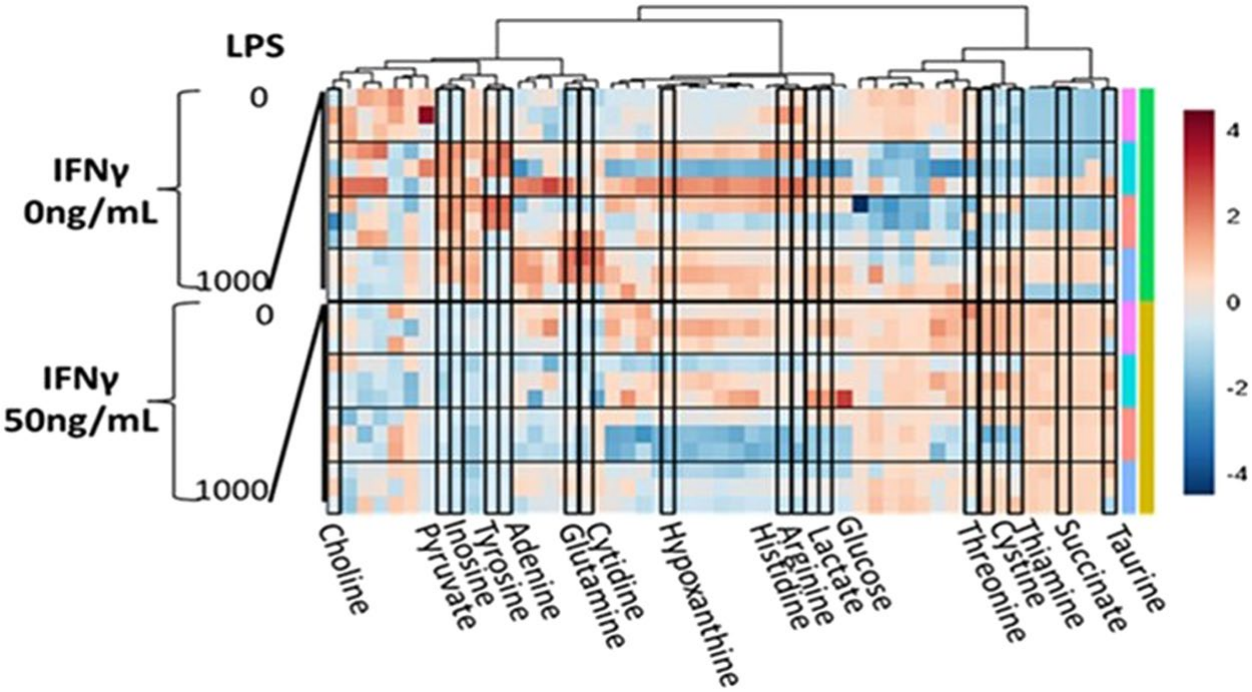
UPLC-MS analysis of extracellular concentration of 54 metabolites. Relative differences in media concentration of 54 metabolites after 24 hr LPS and IFNγ treatment of HUVECs, metabolites forming model bounds highlighted.

Ultra-high pressure liquid chromatography mass spectrometry (UPLC-MS) analysis of spent media after 24 hours of culture defined the baseline metabolism of HUVECs and the perturbations from LPS and IFNγ. Small differences in metabolism between LPS treated and untreated HUVECs were observed (n = 3, two way analysis of variance (ANOVA2) results in Supplement x). These were most apparent at the highest LPS concentration (1000 ng/mL). Aspects of amino acid metabolism, including taurine and glutamine, are altered by IFNγ but not LPS (see detailed statistics in Supplement x). Differences in pyruvate and succinate secretion were observed (Fig. 4 and Supplement x) but the most altered metabolite was inosine. Targeted isotopologue analysis of ^13^C labelled lactate following the addition of 1,2-^13^C glucose confirmed modest changes in glycolytic flux (Supplement xi). The change in distribution of masses of ^13^C labelled lactate were not significant (n = 3, t-test p = 0.530) between untreated and LPS treated HUVECs. This change did, however, translate to a predicted difference in the proportion of lactate produced via glycolysis vs the pentose phosphate pathway in LPS treated cells (68% vs 70%). This prediction is based on the theoretical distribution of labels in a purely glycolytic or pentose phosphate pathway system (Supplement xi). In order to define the inflammatory metabolic perturbations further these data were analysed using iHUVEC2552.

### LPS challenged HUVEC models show differences in glycan and fatty acid metabolism

**Figure 5:**
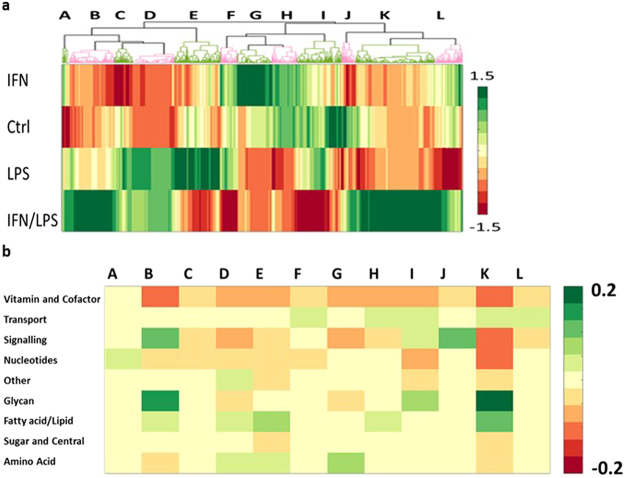
A comparison of the control, LPS, IFN and combined models of HUVEC metabolism. (**a**) Clustering of fluxes with greater than two fold differences across the HUVEC showing the 12 clusters described in the paper. (**b**) The distribution across metabolism (classified by super subsystem) of reactions within each cluster normalized to total reactions of each super subsystem.

We integrated our metabolomics data with publically available transcriptomic data on LPS treated HUVECs^45^. Differences in gene expression and nutrient utilisation of LPS stimulated HUVECs were applied as constraints to reaction flux in iHUVEC2552 (Supplement iii). This process created four models of HUVEC metabolism; control, IFNγ treated, LPS treated, and IFNγ/LPS. To identify endothelial inflammatory metabolic patterns, we compared and analysed the flux space of the GEMs by random sampling (Fig. 5 and Supplement iv).

Glycan metabolism was proportionally the most altered area of metabolism upon stimulation and the third most by absolute number of reactions altered (greater than two-fold change in flux). Fatty acid metabolism was the second most perturbed area of metabolism. Clustering of reaction fluxes across the four HUVEC models showed that the control and IFNγ models clustered together separate from the LPS and IFNγ/LPS models. Greater functional changes in the IFNγ/LPS treated cells are thus reflected in greater metabolic differences. Cluster analysis of reaction fluxes revealed 12 clusters of similarly regulated reactions identified as clusters A through L in Fig. 5 and Supplement v. Most of the clusters contained reactions from various pathways and no distinct pattern emerged. However, cluster B, made up mostly of glycan reactions, was upregulated in the IFNγ/LPS model compared to all other models, indicating replacement of shed glycocalyx in activated cells (Fig. 5). Cluster K that contains many reactions of fatty acid metabolism was least active in the control model, possibly due to membrane restructuring in stimulated cells.

**Figure 6:**
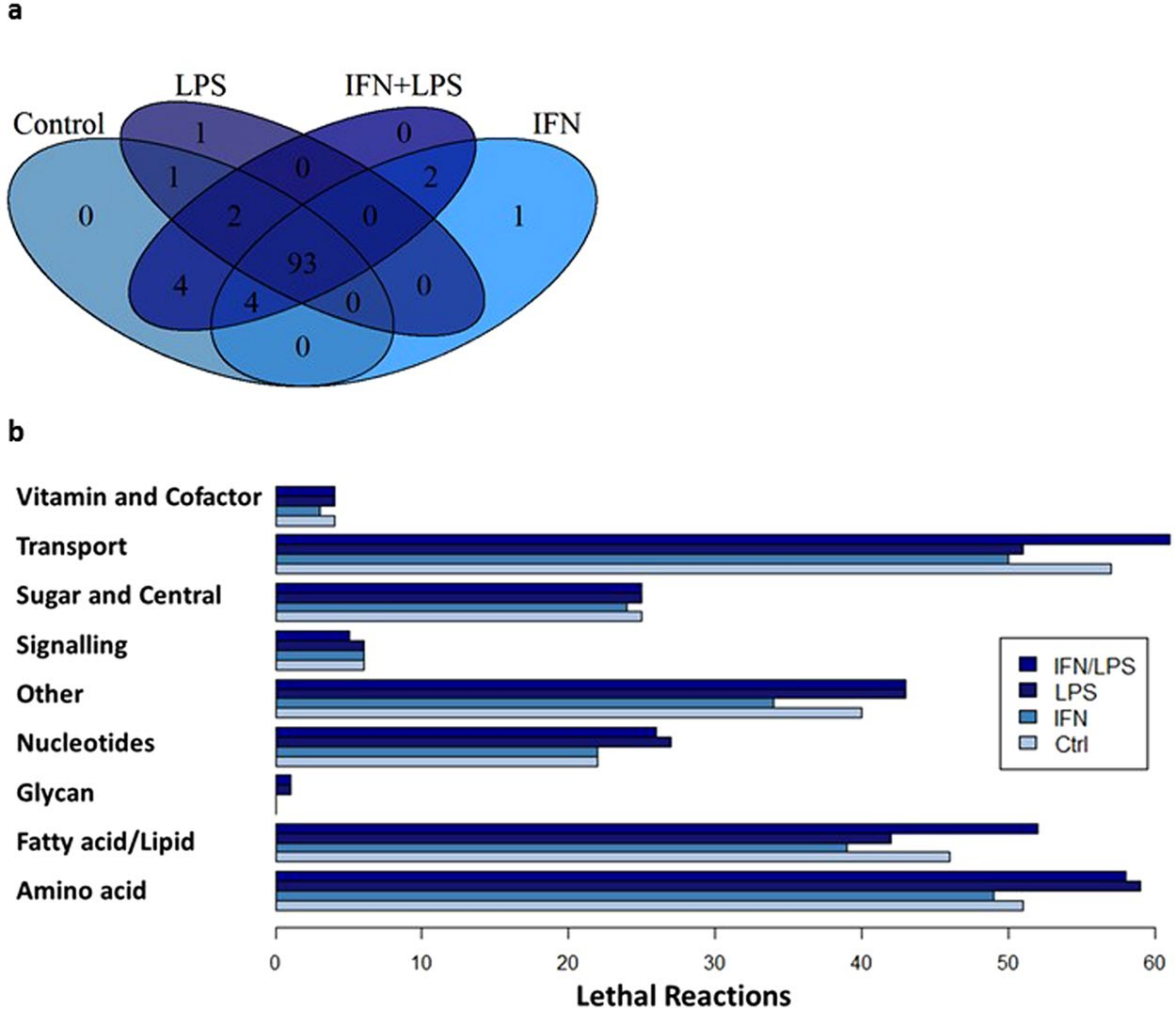
Gene and reaction essentiality in cell culture. (**a**) The shared and unique essential genes of the four cell culture context specific models. (**b**) The distribution by super subsystem of essential reactions in the four context specific models.

We subsequently identified the genes and reactions essential to an active biomass reaction in each model (Fig. 6a,b and Supplement ii). The biomass reaction produces metabolites considered essential for growth and survival^43^. Reactions of transport, fatty acid, and amino acid metabolism were the most common essential reactions. Peripheral and fatty acid metabolism have the most essential reactions unique to one context, indicating that these areas of metabolism differentiate stimulated and unstimulated cells. Inhibiting flux through carnitine palmitoyl transferase inhibited growth in the control model but not in the LPS treated model, suggesting a more robust metabolism with alternatively active fluxes in the LPS model.

In agreement with Patella *et al*.^22^ we found that the control model and the IFNγ model are capable, on optimization, of producing more acetyl-coA from palmitate than from glucose, by around 10%. The LPS and LPS/IFNγ model are equally capable of producing acetyl-coA from palmitate as glucose.

Secretion of biomarkers of endothelial response to LPS or IFNγ stimulation in the HUVEC models were compared to reports of *in vitro* experiments (Supplement iv). Changes in L-kynurenine and methoxy-tryptophan were consistent with literature reported values although changes in NO were at odds with those reported in literature and no reports of changes to GABA, sphingosine-1-phosphate or prostaglandin D2 could be found (Table 2).

**Table 2:** Biomarker mean exchange directions and relative levels compared between control, LPS, IFNγ and combined LPS/IFNγ iHUVEC2552 based models.

Molecule	Control	IFN	LPS	IFNγ/LPS	Match IFN	Match LPS
NO	Secretion	Increase	Decrease	Decrease	NO^83^	NO^40^
S1P	Secretion	Increase	Decrease	Decrease	NA	NA
MTP	Secretion	Decrease	Uptake	Uptake	NA	YES^27^
L-kyn	Secretion	Increase	Increase	Increase	YES^36^	YES^36^
PGD2	Uptake	Uptake	Uptake	Secretion	NA	NA
GABA	Uptake	Uptake	Uptake	Uptake	NA	NA

(NA = no applicable data). Nitric oxide (NO), 5 meth-oxy-tryptophan (MTP), L- kynurenine (L-kyn), sphingosine-1-phosphate (S1P), prostaglandin D2 (PGD2) and gamma amino butyric acid (GABA).

### Sepsis survivor and non-survivor model comparison shows changes to glycan metabolism and agrees with clinical observations of biomarkers

The IFNγ/LPS HUVEC model was modified to account for the differences in plasma metabolite concentrations of septic shock patients from a publically available data-set from Liu *et al*.^35^ Patients were divided into two groups, survivors and non-survivors. These modifications to model constraints (Supplement iii) allowed analysis of metabolic phenotypes of survivors and non-survivors.

**Figure 7:**
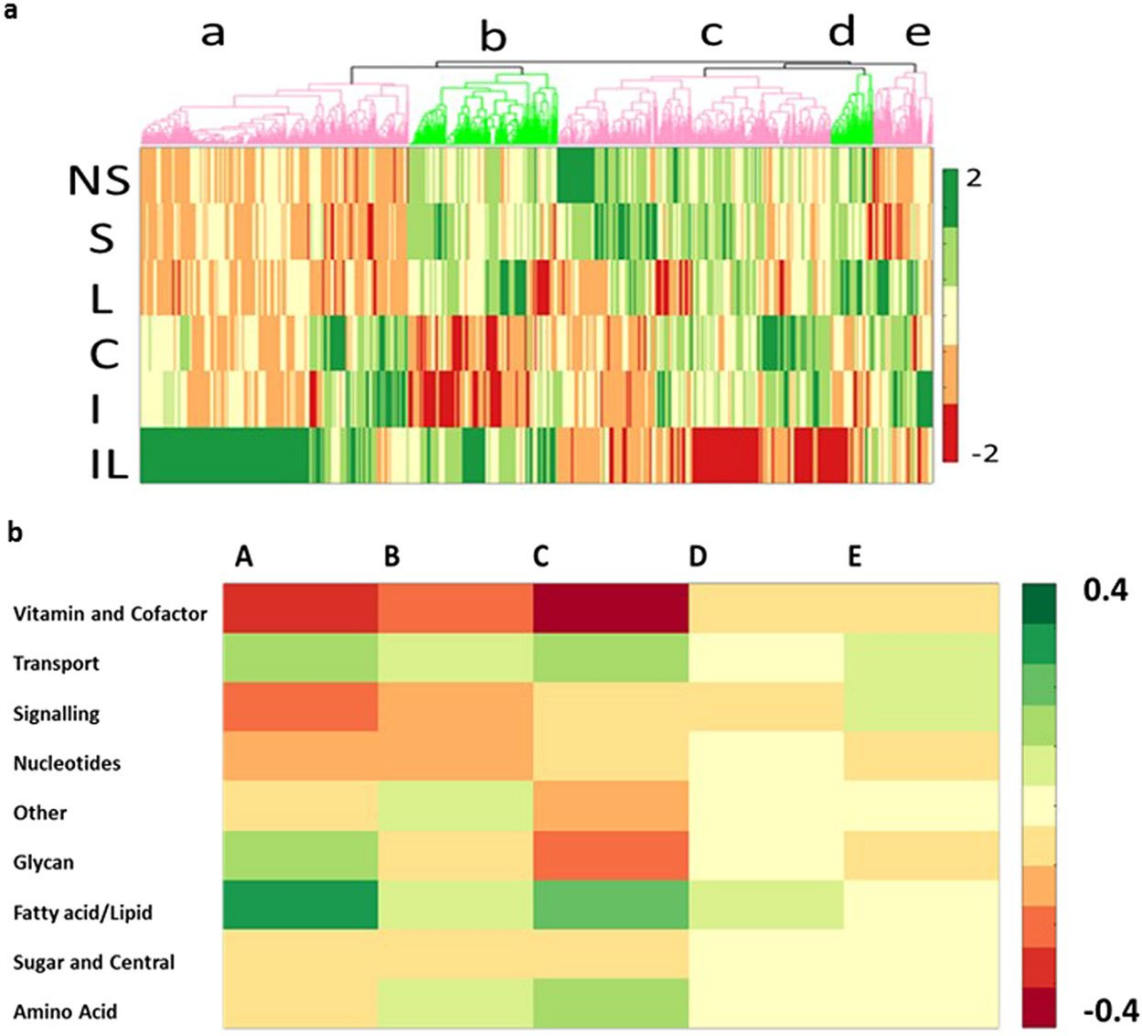
Metabolic changes between sepsis survivors and non-survivors in the context of iHUVEC2552. (**a**) Cluster analysis of 2-fold changes in flux between the two sepsis specific metabolic models and the IFN/LPS cell culture metabolic model (base model). NS: non-survivor, S: Survivor C: Control, I: IFNγ, L: LPS, IL: IFNγ/LPS. Sorted by super subsystem. (**b**) The distribution across metabolism (classified by super subsystem) of reactions within each cluster normalized to total reactions of each super subsystem.

Both patient models were most similar to the LPS treated HUVEC model and had a less severe metabotype than the combined LPS/IFNγ treatment model (Fig. 7a and Supplement v). Fluxes in the non-survivor model were more different from any of the cell culture based models than the survivor model fluxes, suggesting more dysregulated endothelial metabolism. However, the plasma metabolites measured in non-survivors will also reflect metabolic dysfunction in other tissues.

The majority of fluxes altered between survivors and non-survivors were transport fluxes, particularly uptake and secretion fluxes but also mitochondrial transport reactions suggestive of changes to central metabolism (Fig. 7a,b and Supplements v,vi). Glycan metabolism was the second most altered area of metabolism. Fatty acid and amino acid metabolism were also altered, reflecting the changes in the original dataset^35^. The differences in secretion of proline, tyrosine and valine were similar between the control and stimulated HUVEC models as those measured between survivors and non-survivors.

In contrast to what would be expected based on Patella *et al*.^22^ both the survivor and non-survivor models are able to create more acetyl-coA from palmitate than from glucose.

Finally, the secretion of selected endothelial biomarkers by random flux sampling was predicted (Supplement iv). Both models secreted NO, sphingosine-1-phosphate, methoxy-tryptophan, prostaglandin D2, L-kynurenine and GABA albeit to different extent. Increased NO and L-kynurenine secretion and decreased sphingosine-1-phosphate secretion in non-survivors matches reports from previous clinical studies (Table 3)^25,46^. Similarly, the methoxy-tryptophan uptake rate in non-survivors was on average slightly higher than in survivors consistent with reports^27^. However, changes in prostaglandin D2 and GABA in the non-survivor model were not consistent with the changes seen in animal models or patient plasma^38,47^.

**Table 3:** Biomarker mean exchange directions and relative levels compared between sepsis survivor and non- survivor models.

Molecule	Survivor	Non-Survivor	Match
NO	Secretion	Increase	YES^60^
S1P	Secretion	Decrease	YES^84^
MTP	Uptake	Increase	YES^27^
L-Kyn	Secretion	Increase	YES^59^
PGD2	Uptake	Increase	NO^47^
GABA	Uptake	Decrease	NO^38^

Nitric oxide (NO), 5 meth-oxy-tryptophan (MTP), L- kynurenine (L-kyn), sphingosine-1-phosphate (S1P), prostaglandin D2 (PGD2) and gamma amino butyric acid (GABA).

## Discussion

Technical difficulties in sampling primary endothelial cells hinders endothelial research^48^. Sepsis outcomes have improved little in recent decades, possibly due to lack of patient stratification or understanding of disease biology^29^. Endothelial dysfunction in sepsis contributes to disease severity and is characterised by the loss of surface glycocalyx and increased permeability^10^. To increase our understanding of the complex relationship between sepsis and endothelial metabolism we created the endothelial specific metabolic model iEC2812, thus allowing the investigation of sepsis patient endothelial metabotypes.

iEC2812 and the sub-type specific reconstructions are based on a curated version of RECON1 and allow the integration of transcriptomic and metabolomics data. Manual curation of RECON1 ensured that the links between genes and reactions were improved making the reconstructions better able to account for metabolic features of endothelium. In particular, we addressed the production of L-kynurenine and methoxy-tryptophan as well as reducing dead-ends in chondroitin and heparan sulphate metabolism.

All the models were able to produce six key endothelial biomarkers; NO, methoxy-tryptophan, L-kynurenine, sphingosine-1-phosphate, prostaglandin D2 and GABA. NO is a vascular relaxing factor^40^. Methoxy-tryptophan is an anti-inflammatory factor secreted and responded to by endothelial cells^27,49^. L-kynurenine is another tryptophan metabolite that has been shown to play a role in sepsis and to be produced by endothelial cells^26,36^. Sphingosine-1-phosphate is a bioactive lipid which acts on sphingosine-1-phosphate receptors to mediate various cardiovascular and immune effects^50–53^. Prostaglandins are produced from arachidonic acid and are important inflammatory signalling molecules which are secreted by the endothelium^39^. Although our models produce and secrete prostaglandin D2, they do not secrete prostaglandin E2, a more endothelial specific prostaglandin. This is despite the fact that they have the structural ability to do so. GABA, a product of glutamic acid metabolism is a well-known neuro-transmitter that has recently been identified as a vascular inflammatory mediator^38^. These features make iEC2812 a fuller representation of known endothelial metabolic response to inflammatory stimuli than RECON1 and the HUVEC GEM published by Patella *et al*.^22^. However, many of the metabolites are under post transcriptional control and therefore may need more specific metabolomic data to describe their production accurately.

An important part of this study was determining an appropriate endothelial subtype to serve as a model to study sepsis metabolism or endothelial dysfunctional metabolism *in vivo*. We generated three endothelial sub-type models, each based on a data set containing transcriptomic data from multiple individuals^54^. This produced sub-type specific models of that showed that metabolism of these endothelial sub-types are similar. This is important as access to most endothelial sub-types is limited and HUVECs represent the most easily accessible endothelial cell type. These results conform to the original paper that highlighted similarity in the transcriptome of these endothelial sub-types^54^. We found that around 90% of genes essential for the biomass reaction are shared as around 90% of metabolic genes overall are shared. The essential genes shared by the sub-type models included genes that are known to be important to endothelial function (Supplement ii).

To more accurately model the metabolism of endothelial dysfunction in sepsis, we stimulated HUVECs with LPS *in vitro* and measured extracellular metabolites using UPLC-MS. LPS is commonly used as an *in vivo* sepsis model^17^. Co-stimulation with LPS and IFNγ has been shown to sensitise endothelial cells to LPS^19^. Our results were consistent with previous reports despite being obtained from a fairly small sample size which may reduce the validity of the conclusions, especially given that they are from primary cells where individual variation may play an important role. We found differences in amino acid metabolism including tryptophan metabolism, significant with IFNγ, which is important to several of the biomarkers which we assessed. Overall, HUVEC metabolism is fairly robust with respect LPS stimulation, although it shifts slightly further when LPS and IFNγ are combined.

In parallel, we performed functional studies to examine the effects on endothelial function after LPS and IFNγ stimulation. The experiments indicated that the glycocalyx was shed in response to stimulation with LPS or IFNγ alone and to a greater extent in response to combined stimulation. It has been shown that higher concentrations of glycocalyx components are found in the plasma and urine of sepsis patients than healthy controls indicating that the glycocalyx is shed during sepsis^55,56^. In particular, heparan sulphate proteoglycan 2, shown in red in Fig. 3b, has been shown to appear in the urine of patients at a higher rate than healthy individuals^55,56^. Endothelial permeability increased with LPS and IFNγ which indicates that the experimental system reproduces to some extent the clinical effects of sepsis, namely vascular leakage and loss of glycocalyx (Fig. 3d).

The metabolimic data was integrated into iHUVEC2552 to create context specific models of inflammatory stimulation of HUVECs. These models showed increasing metabolic perturbation with increasing inflammatory stimulation. In particular, the production and processing of glycans was upregulated in the LPS/IFNγ stimulated model. This coincided well with the immunofluorescent images showing less glycocalyx in the LPS/IFNγ stimulated cells than in either of the separately stimulated samples. Heparan sulphate proteoglycan 2, a glycocalyx component found to be increased in the urine of sepsis patients experiencing capillary leakage, was also lost during LPS and IFNγ stimulation of HUVECs^55,56^ (Fig. 3a–c).

Our models predict that untreated and IFNγ treated cells are able to produce more acetyl-coA from palmitate than from glucose while LPS treated while LPS/IFNγ treated cells do not. Patella *et al*. reported greater production of acetyl-coA from fatty acids than from glucose in intact endothelium, our *in vitro* data show that LPS treated cells are more permeable than untreated HUVECs (Fig. 2d) suggesting that this is a functionally relevant aspect of the model, consistent with previous findings^22^.

Our analysis of six selected biomarkers was broadly concordant with the literature (Table 2). This suggests that although these metabolites are under control of various signalling pathways, metabolic alterations influence their uptake and secretion and are, for the most part, well represented in these models. This modelling effort allowed us to contextualise the functional data we produced and to predict changes in glycocalyx metabolism that were not included in our metabolic data set. There are also other changes to metabolism predicted by the model that warrant future investigation, for example alterations to pyrimidine metabolism (Supplement iv and v). These have not been confirmed by literature search or experimentally, however they provide directions for future research. The IFNγ model was based on the same transcriptomic data as the untreated model. Therefore, differences between IFNγ and untreated models and similarities between IFNγ and LPS models are emergent model properties. This means that results that agree with the current knowledge base provide reassurance about the model validity and novel results provide hypothesise that can be tested in future studies.

Having verified that the models correspond with the *in vitro* effects of inflammatory stimuli on endothelial cells, we applied the models to the meta-analysis of plasma metabolomics data regarding sepsis survival^35^. Plasma contains metabolites secreted and taken up by multiple cell types. However, given that the endothelium is in close contact with blood it is likely that to be a large contributor to the plasma metabolome. This is supported by several changes reported in the initial sepsis study^35^, qualitatively matching changes in the models. The data-set from Liu *et al*.^35^ highlighted amino acids and fatty acids as differentiating sepsis survivors from non-survivors. Many of these amino acid changes were replicated in our models. Fatty acid metabolism was more perturbed in the non-survivor model than in the survivor model, also consistent with the data from Liu *et al*. However, we did not see the expected increase in production of acetyl-coA from glucose compared to palmitate in either model as reported by Patella *et al*.^22^. Glycan metabolism was highly altered in the non-survivor model. This is consistent with glycocalyx shedding which predicts sepsis outcome as increased free syndecan-1, a heparan sulphate protein, in plasma indicates poor prognosis in sepsis^57^.

The biomarker profiles of sepsis survivors were different from non-survivors, despite none of these being model constraints (Table 3). Sphingosine-1-phosphate is known to increased barrier integrity in endothelium. Therefore, lower plasma levels may be deleterious and predictive of poor outcome in sepsis^25,58^. Our models predicted lower secretion of sphingosine-1-phosphate in non-survivors. Methoxy-tryptophan is an anti-inflammatory molecule, higher levels of which are associated with sepsis survival in a murine model^27^. In accordance with these data, our patient models showed greater uptake of methoxy-tryptophan in non-survivors resulting in a lower plasma concentration. The secretion of both NO and L-kynurenine have been linked to catastrophically reduced blood pressure in sepsis. It is therefore expected that they are increased in non-survivors over survivors, this is also predicted by our models^59,60^. However not all of the predicted changes were in accordance with the data, for example the changes to prostaglandin D2 data did not agree with expectations. There is predicted to be an uptake of prostaglandin D2 that is highest in non-survivors, leading to lowest predicted plasma concentration in the model. However patients who do not survive sepsis have been shown to have higher plasma prostaglandin D2 than those who do^47^. This may be because prostaglandin D2 synthesis is a process in which many other cell types influence plasma concentration and there is a high degree of post-transcriptional control which would not be expected to be fully accounted for by the model.

The models and the predictions they provide will only be as good as the data used to create them. The fact that endothelial cells of different origin converge with respect to phenotype after multiple passages may reduce the reliability of the transcriptomic data. However the data we used is from cells at a lower passage number than is associated with this issue^48,61^. Furthermore, RNA transcription is not absolutely correlated with protein abundance and enzymatic activity^47^.

The biomass function used to predict essential genes and reactions in these analyses is the same for all models and may therefore underestimate biological differences^43,48^. It is important to state that the conclusions we have drawn from the patient derived models (survivors versus non-survivors) are limited by the context of the *in vitro* experiments. The *in vitro* experiments and therefore the GEMs do not include the many types of inflammatory cell and their signalling pathways involved in human sepsis. This lack may be particularly important with respect to lipid inflammatory mediators which may be detected in the plasma of sepsis patients. The case of prostaglandin D2 highlights the limitations of this modelling approach as it does not properly account for metabolic changes due to short term signalling. Further work *in vitro* and *in vivo* is necessary to fully assess the impact of complex signal cascades mediated by other cell types in this context. Nonetheless our models are in agreement with many previously described aspects of endothelial metabolism in sepsis and may be used it to further our understanding of endothelial dysfunction.

Our analysis of context specific models has demonstrated the critical role of glycan metabolism and confirmed alterations in fatty acid metabolism in endothelial inflammatory activation. We have also shown that patients who survived sepsis had more metabolic features shared with less activated endothelium. The endothelial biomarker profiles predicted by our models broadly corresponded with the changes expected upon endothelial dysfunction caused by sepsis. This demonstrates that analysing plasma metabolic data from patients with an endothelial GEM provides results with practical relevance. The metabolic models are available as a resource to study the many other critical roles which endothelial cells play in human disease.

## Materials and Methods

### Cell culture

Primary HUVECs, obtained from Landspitali University Hospital, Reykjavík, Iceland, were harvested by a modification of the method of Jaffe *et al*. as previously reported^62,63^. The cells were harvested by collagenase digestion and seeded in 25 cm^2^ flasks with 5 mL of Endothelial Growth Medium (EGM) (Lonza, Basel, Switzerland), containing 2% foetal bovine serum and antibiotics (penicillin, 100 units/mL and streptomycin, 100 μg/mL Thermo-Fisher Scientific, Waltham, USA). Cells were incubated at 37 °C in humidified air with 5% CO_2_. Medium was changed 24 hours after seeding and every 48 hours thereafter confluence was reached (~5 days), they were then used or sub-cultured. For use cells at passage 1–3 were seeded onto 35 mm^2^ culture plates in 2 mL EGM. At confluence control media or media containing 50 ng/mL IFNγ (R&D systems, Minneapolis, USA), was added and the cells were incubated for 3 hours. Then control media or 10 ng/mL, 100 ng/mL or 1000 ng/mL LPS was added (*E*. *coli* O111:B4 Enzo Lifesciences, Farmingdale, USA) and incubated for either 4 or 24 hours. After incubation the cells and media were collected and stored at −80 °C prior to analysis. These experiments were performed in accordance with approval granted by the National Bioethics Committee of Iceland and Icelandic Data Protection Authority, following their guidelines on informed consent.

### Isotope labelling

HUVECs were cultured to confluence as above and then had their medium changed to EGM plus with added 5 mM normal glucose or 5 mM 1,2-^13^C labelled glucose (Cambridge Isotopes, Tewksbury, USA) with and without 10 ng/mL LPS. Medium was collected as above after 4 hours and 24 hours incubation with ^13^C glucose.

### Mass Spectrometry

UPLC-MS Analyses were performed with an UPLC system (UPLC Acquity, Waters, Manchester, UK) coupled in line with a quadrupole-time of flight hybrid mass spectrometer (Synapt G2, Waters, Manchester, UK) as described in Paglia *et al*.^64^. Additional glucose and lactate measurements were performed in an ABL 90 gas analyzer (Radiometer, Brønshøj, Denmark). Missing values were replaced by the minimum positive values detected for each parameter. The data was first batch normalized by dividing each variable of each batch by the square root of the sum of squares of original values of that batch. We then used a standard metabolomics analysis pipeline following the MetaboAnalyst online tool^65^. This dataset was normalized by constant sum, log transformed and scaled using unit variance scaling method (mean-cantered and divided by the standard deviation of each variable). Data normalisation and heat mapping of metabolites measured in medium of HUVECs and a comparison of extracellular metabolites by were performed in the online Metaboanalyst tool^65^ using heat mapping for data visualisation and a two way ANOVA test ANOVA (Two-way (between subjects) ANOVA with type I, weighted means) with false discovery rate correction to assess the effects and interaction of the LPS and IFNγ treatment on HUVEC metabolism. For isotopologue analysis, IsoCor^33^ corrected for natural isotope abundance providing the percentage of heavy isotopes that exceed natural abundance and the corrected isotopologue distribution.

### Fluorescent imaging of HUVECs treated with IFNγ and/or LPS

HUVECs were seeded at 100 000 cells/ml in 400 µl of media into an 8 well chamber slide (80841 Ibidi, Planegg, Germany) coated with gelatine. The cells settled for 72 hours. The media was changed 24 hours to remove unattached cells. After 72 hours (at confluence) the media was replaced with control media or media containing 50 ng/mL IFNγ and incubated for 3 hours, this was then removed and replaced with control or 10, 100, or 1000 ng/mL LPS containing media and incubated for 4 hours. This media was discarded and the cells washed with phosphate buffered saline and then fixed with 10% formalin. Cells were then blocked with 5% bovine serum albumin (BSA), on ice, before staining with 1:100 dilution of mouse anti-heparan sulfate anti-body (Anti-heparan sulphate proteoglycan 2 antibody [A74] ab23418 Abcam, Cambridge, UK) in BSA and goat anti-mouse secondary anti-body conjugated to Alexa fluor 555 (Goat anti-mouse IgG(Alexa Fluor 555) ab150114, Abcam, Cambridge UK) at a 1:300 in BSA dilution then wheat germ agglutinin 5 µg/mL diluted in PBS (Alexa Fluor 488 conjugate W11261, Thermo-Fisher Scientific, Waltham, USA), and finally Fluoromount-G diamidino phenylindole (DAPI) (eBioscience Affeymetrix, Vienna, Austria). Cells were imaged using a Leica DMI6000B microscope and CTR 6500 electronics box (Leica Microsystems, Wetzler, Germany) with an EXi Blue Fluorescent Microscopy Camera (QImaging, Surrey, British Columbia, Canada) controlled by Micro-Manager software^66^. Readings were made at excitation wavelength of 555 nm, emission wavelength 565 nm to image the heparan sulphate proteoglycan 2, excitation wavelength of 495 nm, emission wavelength 519 nm to image the wheat germ agglutinin, and excitation wavelength of 340 nm, emission wavelength 488 nm to image DAPI. Quantification of the fluorescent areas was performed using ImageJ software^67^ and normalized to area. Results were analysed using a two sample two tailed equal variance t-test and considered significant at p < 0.05.

### Endothelial Permeability Assay

Endothelial permeability was estimated by measuring the flow of fluorescently labelled dextran- fluorescein isothiocyanate–dextran (FITC)(70 000 dalton, Sigma-Aldrich/Merck-Milipore, Darmstadt, Germany)^68^. HUVECs were seeded onto Transwell cell culture inserts (4 µm pore, 12 mm diameter, Sigma-Aldrich/Merck-Milipore, Darmstadt, Germany) coated with 0.1% gelatine. HUVECs were grown to confluence (~72 hours), as above. Media was then removed and replaced with either control media or media containing 50 ng/mL IFNγ and incubated for 3 hours. Cells were then stimulated with 1000 ng/mL LPS for 4 hours the medium in the upper chamber was then removed and replaced with EGM containing FITC-dextran at 1 mg/ml (Sigma) and LPS. Tumour necrosis factor α (Bio-Techne Ltd, Abingdon, UK) was used as a positive control. The plate was incubated for a further 45 minutes and medium from the lower chamber assayed for fluorescence at excitation wavelength of 488 nm and emission wavelength of 520 nm. Results were analysed using a two sample two tailed equal variance t-test and considered significant at p < 0.05.

### Endothelial model reconstruction

An overview of the reconstruction and constraint is shown in (Fig. 1). iEC2812 is a genome scale metabolic reconstruction of endothelial cells based upon RECON 1^42^. RECON1 is a comprehensive genome scale metabolic reconstruction of humans. A genome scale metabolic reconstruction is a stoichiometric matrix representing metabolic reactions. This is linked to gene rules describing the genes that need to be transcribed to produce the enzymes needed for each reaction. This allows the integration of genomic or transcriptomic data with metabolic data. To create iEC2812, transcriptomic data from ArrayExpress (E-GEOD-21212) was used to prune RECON1^54,69,70^ using Fastcore^71^ to obtain a consistent model which was then manually curated to include reactions of tryptophan metabolism and extra transport reactions from RECON2^72^ (Supplement i) using the Cobratoolbox in Matlab (Mathworks, Natick, Massachusetts, USA)^73^. The Affymetrix IDs in the dataset were converted to Entrez IDs using DAVID^74^ converter to enable integration with the gene reaction rules in the reconstruction^74,75^. All transcripts not called as absent in the combined gene-set were compiled into a single endothelial list and used with the Fastcore algorithm in Matlab to create an overall model of endothelial cell metabolism i.e. iEC2812^71^. The data set used cells from multiple individuals and should, therefore, be representative of the population.

### Generation of endothelial specific Genome Scale Metabolic Models

An identical process was followed with each of the three separate gene-sets (datasets GSM530359-62 combined (HUVEC), GSM530363-66 combined Human microvascular endothelial cells (HMVEC), GSM530371-74 combined human pulmonary artery endothelial cells (HPAEC))^54^ to determine the active reactions in each sub-type reconstruction. Endothelial subtype specific GEMs were further constrained by assigning which reactions were up and down regulated in each cell type as compared to the HUVEC gene-set using the expression values in the data set and a cut-off (+/−1.5) that ensured that no gene occurred in both up and down regulated sets. Constraints on these reactions were then multiplied by 2 or 0.5 respectively. This value was chosen as it was close to the mean change up or down in the transcript levels. Additional endothelial subtype specific metabolic flux constraints were then added manually based on data from the literature, see (Supplement iii).

In order to produce models of control, LPS stimulated, IFNγ stimulated and LPS/IFNγ stimulated HUVEC cells. iHUVEC2552 was further constrained using publically available transcriptomic data, (ArrayExpress data set E-GEOD-50619 sub-set GSM1224743^76^) descriptive of transcriptomic changes in cultured HUVECs following LPS stimulation, in addition to our metabolomics. Affymetrix IDs were converted to Entrez IDs using DAVID then genes separated into up and down regulated genes (untreated sample as baseline). This produced a new base model that was used to create the LPS and LPS/IFNγ combination treated models while the original model was used as the base for a control model and the IFNγ treated HUVEC model. Following this constraints on uptake and secretion reactions were applied based on metabolomics data from the medium of HUVECs cultured as above (control, LPS treated, IFNγ treated and LPS/IFNγ treated). Uptake and secretion of 20 metabolites and data from cells grown with 1,2-^13^C labelled glucose to constrain the ratio of flux through the reactions of glycolysis vs pentose phosphate pathway (Supplement iii). These constraints were based on the mean data of three repeats with bounds being 10% above and below the mean for each group to allow for the possible greater differences than seen in the current experimental procedure. This further constraining of the models used the change reaction bounds function in Cobratoolbox in Matlab^73^. A minimal number of other exchange fluxes were relaxed in order to obtain a feasible model. This created control and treated models.

### Application of sepsis survivor and non-survivor metabolic data to iHUVEC2552

A literature screening of publically available metabolomics based analyses of sepsis patients^15,16,35,77,78^ identified one study^35^ that included time points that overlapped with our cell culture experiments and containing data amenable to analysis using iHUVEC2552. Metabolomics data from this study of sepsis survivors and non-survivors was used to constrain the LPS/IFNγ model. Exchange fluxes in iHUVEC2552 relating to metabolites in the data set were identified. The minimum and maximum flux of these exchange reactions, determined by random sampling of the LPS/IFNγ model, became the upper and lower bound of these reactions for the survivor model. If these fluxes were 0 then constraints of 0–100 was assumed. The exchange reaction bounds for the non-survivor model took the survivor model as a base. The fold change of the metabolites reported in the paper was applied to the bounds of the exchange fluxes in the survivor model to create the non-survivor model for example a metabolite with a fold change of 5 with survivor model bounds of 1–2 would have bounds of 5–10 in the non-survivor model (constraints on exchange fluxes in Supplement iii).

### Model analysis

The models were analysed using flux balance analysis, flux variability analysis, random sampling, and essential reaction and gene features (reactions essential for the biomass reaction to be active) in the Cobratoolbox in Matlab^73,79^. Further analysis of these data was performed in Matlab using two sample two tail t tests with Bonferroni correction and significance at p < 0.05, to determine if reactions had significantly different predicted fluxes between stimulated and unstimulated models and the heatmap and clustering functions to group reactions, the spearman pairwise distance value was calculated within the clustergram function in Matlab to form the clustergrams. VennDiagram^80^, plotrix^81^ and ggplot packages^82^ in R (R Development Core Team, Vienna, Austria) were used for data visualisation.

### Data Availability Statement

Data is available in the supporting information or upon application to the authors.

### Disclosures

Umbilical cord vein sampling was approved by the National Bioethics Committee of Iceland and Icelandic Data Protection Authority.

## Electronic supplementary material

Supplement I

Supplement II

Supplement III
