# Supplementary material for: Metabolic systems analysis of LPS induced endothelial dysfunction applied to sepsis patient stratification
Source: Sci Rep. 2018 May 1;8:6811. doi: 10.1038/s41598-018-25015-5 (PMC5931560; doi:10.1038/s41598-018-25015-5)
Supplement: Supplementary file 2 — Supplement II [file 41598_2018_25015_MOESM2_ESM.pdf]

Metabolic systems analysis of LPS induced endothelial dysfunction applied to sepsis patient stratification

Sarah McGarrity[1#], Ósk Anuforo[1], Haraldur Halldórsson[2,3], Andreas Bergmann[1], Skarphéðinn Halldórsson[1], Sirus Palsson[1], Hanne Hee Henriksen[4], Pär Ingemar Johannsson[1,4+] and Óttar Rolfsson[1,2*+].

[1]Center for Systems Biology, University of Iceland, Sturlugata 8, Reykjavik, Iceland.

[2]Medical Department, University of Iceland, Sturlugata 8, Reykjavik, Iceland.

[3]Landspitali, Læknagarður, Hringbraut, Reykjavik, Iceland.

[4]Rigshospitalet, Blegdamsvej 9, 2100 København Ø, Denmark

#First Author

+Joint Senior Author

***Corresponding Author**

Correspondence Address: Dr Óttar Rolfsson, Center for Systems Biology, University of Iceland, Sturlugata 8, Reykjavik 101, Iceland. Email: ottarr@hi.is Telephone: 00354 525 5854, Fax: 00354 552 1331

Supplement II

a *Assessment of iHUVEC2812*

b *Comparing HUVEC, HPAEC and HMVEC models*

c *Functional and metabolomics analysis of LPS and IFNγ stimulation of HUVECs*

d *^{13}C isotopologue analysis*

Supplement IIa – Assessment of iHUVEC2812

We built a metabolic reconstruction of EC metabolism by curating RECON1 then using Fastcore[1] to apply a transcriptomic data-set. Removal of inconsistent reactions by Fastcore, those that are dead ends or otherwise unconnected in the model, removed 34 % (1,270/3,743) of reactions in RECON1 but less than 14% (301 reactions) in the HUVEC model based on iEC2812 before application of metabolic constraints and provides a measure of the influence of literature curation.

Roughly 80 % of nucleotide and amino acid metabolism reactions are retained. There are extra reactions added in fatty acid and signalling metabolism. Around 70 % of reactions of sugar and central carbon reactions are retained. Whereas only around 60 % the reactions classified as peripheral metabolism (other), vitamin metabolism and exchange reactions were retained (**Figure 1**).

In comparison to RECON1[2], iEC2812 had a higher proportion of reactions linked to genes, around 70 % compared to around 60 % in RECON1[2]. Also of the genes present in the model only around 50 % in RECON1[2] were used whereas in iEC2812 around 70 % of genes were used **(Table1)**.

Supplement IIb *–* ***Comparing HUVEC, HPAEC and HMVEC models***

To determine if and how metabolism differed between three endothelial subtypes, commonly used in laboratory research, we built three GEMs based upon iEC2812.

These are slightly more extensive than a previously published model of HUVEC metabolism containing ~2,550 reactions **(Table1)** compared to 2088 active reactions[3]. This is partly due to using transcriptomic rather than proteomic data to constrain reactions and partly due to the improved base model.

Analysis of reactions and genes essential for survival showed roughly 90 % of essential reactions were common to at least two reconstructions, this is partly a reflection of the composition of the biomass function. Glycerophosphlipid metabolism, nucleotide metabolism and sphingolipid metabolism were strongly represented in all sets of essential reactions. Essential reactions found only in HUVECs were in nucleotide and nucleotide sugar reactions. Essential reactions only in HPAECs four are reactions of nucleotide metabolism or transport, one is a ceramide reaction, and the other three are amino acid metabolism or transporters. Essential reactions only in HMVECs include nucleotide metabolism and transport reactions, and an amino acid transport reaction. On the other hand common essential reactions include cardiolipin synthase 1, phosphatidylglycerophosphate synthase 1 and sphingomyelin synthase 1 all of which have previously been associated with cardiovascular disorders[4,5] **(Figure1)**.

The ability of iEC2812 and sub-type models to describe known features of endothelial metabolism were queried by their ability to secrete known biomarkers of human endothelium. Nitric oxide, meth-oxy-tryptophan, kynurenine, spingosine-1-phosphate, prostaglandin D2 and prostaglandin E2 and gamma amino butyric acid have all been previously shown to be secreted by ECs and function in signalling both by and to ECs[6–11].

Supplement IIc- Functional and metabolomics analysis of LPS and IFNγ stimulation of HUVECs

Wheat germ agglutinin normalised to area Fold change and p-values for cells with different treatments.

IFN	LPS(ng/mL)	Mean	SD	Change v blank control	Change v No IFN	pvalue v blank control	pvalue v No IFN
No IFN	***0***	0.15	0.02	NA	NA	NA	NA
	10	0.14	0.03	-7%	NA	0.414	NA
	100	0.13	0.01	-13%	NA	0.145	NA
	1000	0.11	0.01	-27%	NA	0.001**	NA
IFN	***0***	0.13	0.02	-13%	-13%	0.163	0.163
	10	0.14	0.04	-7%	0%	0.519	0.999
	100	0.12	0.01	-20%	-8%	0.011*	0.157
	1000	0.09	0.02	-40%	-18%	0.001**	0.211

*= p<0.05 **= p<0.01

Heparan sulfate normalised to area Fold change and p-values for cells with different treatments.

IFN	LPS (ng/mL)	Mean	SD	Change v blank control	Change v No IFN	pvalue v blank control	pvalue v No IFN
No IFN	***0***	0.25	0.03	NA	NA	NA	NA
	10	0.19	0.06	-24%	NA	0.117	NA
	100	0.19	0.04	-24%	NA	0.041*	NA
	1000	0.13	0.02	-48%	NA	p<0.001**	NA
IFN	***0***	0.17	0.05	-32%	-32%	0.023*	0.367
	10	0.16	0.05	-36%	-16%	0.021*	0.449
	100	0.15	0.02	-40%	-21%	0.001**	0.271
	1000	0.11	0.03	-56%	-15%	p<0.001**	0.266

*= p<0.05 **= p<0.01

Supplement IId – ^{13}C isotopologue analysis

Table IIIdA- Results of two way ANOVA from metaboanlyst for 24 hour extracellular metabolites from HUVECs

**Name**	**IFNg**<br>**FDR corrected P value 2 way ANOVA**	**LPS concentration**<br>**FDR corrected P value 2 way ANOVA**	**Interaction**
**Inosine**	2.0624E-6	0.015918	0.0074712
**Malate**	3.5372E-5	0.015918	0.0074712
**Aconitic acid**	0.0014336	0.015918	0.014778

Name	IFNg<br>FDR corrected P value 2 way ANOVA	LPS concentration<br>FDR corrected P value 2 way ANOVA	Interaction
**Pyruvate**	4.6287E-6	0.050706	0.015321
**Methionine**	0.013157	0.60195	0.06284
**Cysteine**	0.019528	0.18721	0.094117
**ADMA**	0.0014336	0.32213	0.094521
**Fumaric acid**	0.0017162	0.035836	0.097988
**Cystine**	0.022519	0.18721	0.19132
**Adenine**	0.025174	0.29977	0.30048
**Tryptophan**	2.8182E-7	0.73821	0.30048
**Glyceric acid**	0.21167	0.043128	0.31864
**Tyrosine**	0.019528	0.56294	0.46836
**Valine**	0.0045795	0.58661	0.46836
**Cytidine**	0.019528	0.18658	0.59983
**Succinate**	0.0017119	0.60195	0.59983
**Uridine**	0.0014336	0.62926	0.59983
**Glutamate**	2.6261E-4	0.60195	0.60623
**Arginine**	0.02006	0.42664	0.84955
**Taurine**	0.025416	0.60195	0.96683

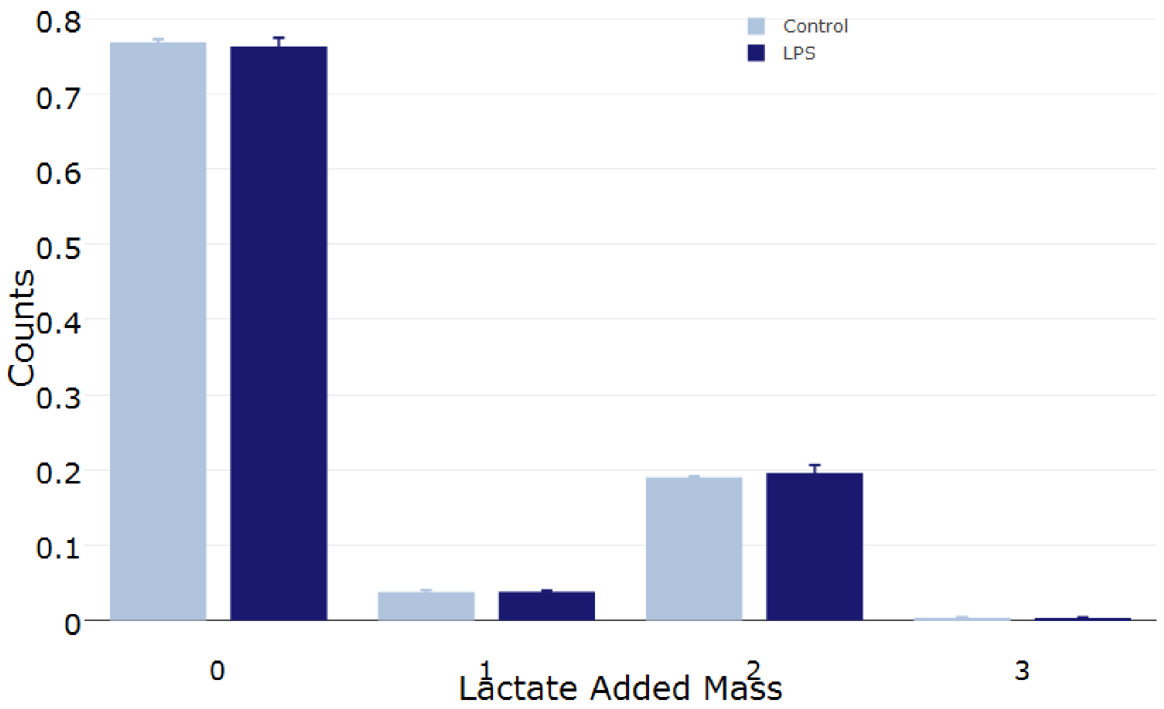

A Mass distribution of lactate after 24 hours growth with 1,2 ^{13}C glucose and LPS. *^{13}C isotopologue analysis of lactate after 1,2-^{13}C glucose treatment*

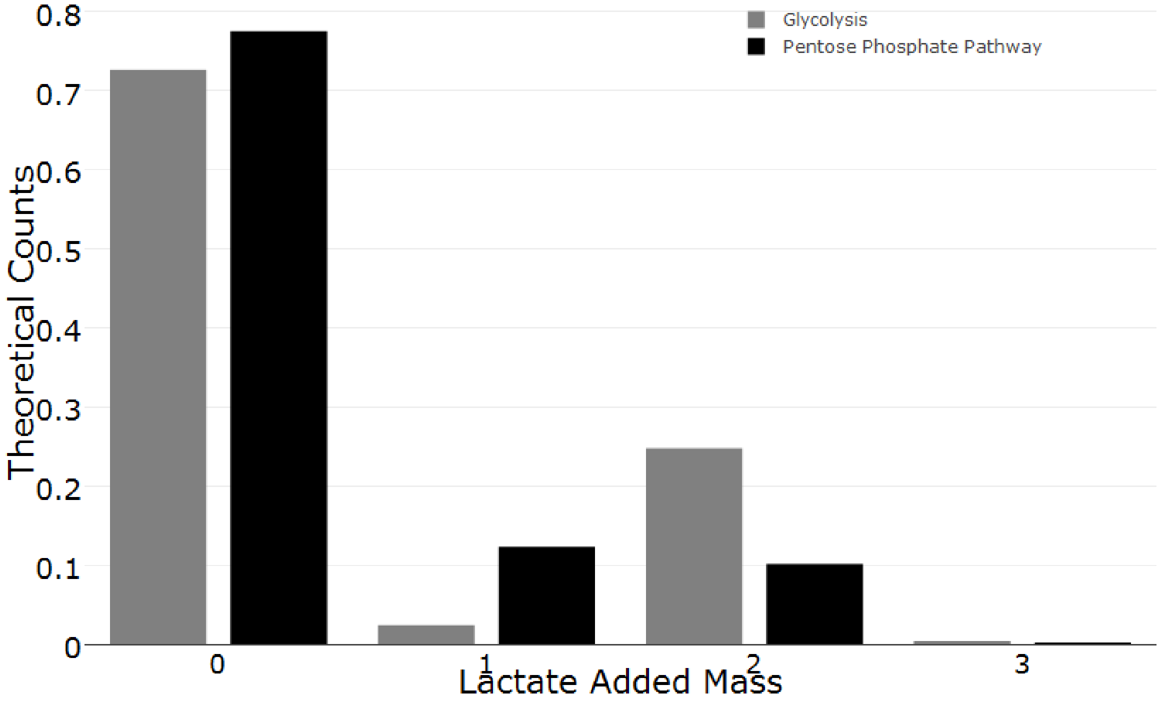

B Theoretical mass distribution *of lactate in glycolysis and pentose phosphate pathway after 1,2-^{13}C glucose treatment*

The mass distribution of extracellular lactate was determined using UPLC-MS followed by Iso-core processing after growth with 50% 1,2^{13}C labelled glucose (A). It

was confirmed that the overall glucose consumption/lactate secretion rate was unaffected by the extra glucose. It was also shown that there was no significant difference in mass distribution values between 0,1,2 or 3 labelled lactate with and without LPS after 24 hours. On t-tests of the proportion of each mass of lactate mass+0 $p = 0.568$, mass+1 $p = 0.878$, mass+2 $p = 0.487$, mass+3 $p = 0.680$ and the overall mean enrichment of distribution value was 0.143 in control cells and 0.146 in LPS treated cells ($p = 0.530$).

This distribution was compared, using linear least squares fitting in Matlab, to the theoretical mass distribution of lactate in cells if all lactate was produced either via glycolysis or via the pentose phosphate pathway (B). This suggests a mean of 68% glycolysis in control cells and 70% in LPS treated cells, this small difference is in line with the non-significant difference seen in the mass distributions, and is reflected in the constraints placed on the models shown in **Supplement Ib**.

References Supplement II

1. Vlassis N, Pacheco MP, Sauter T, Miller J, Kisters-Woike B. Fast Reconstruction of Compact Context-Specific Metabolic Network Models. Ouzounis CA, ed. *PLoS Comput Biol*. 2014;10(1):e1003424. doi:10.1371/journal.pcbi.1003424.
2. Duarte N, Becker S, Jamshidi N, et al. Global reconstruction of the human metabolic network based on genomic and bibliomic data. *PNAS*. 2007;104(6):1777-1782. doi:10.1073/pnas.0610772104.
3. Patella F, Schug Z, Persi E, et al. Proteomics-Based Metabolic Modeling Reveals That Fatty Acid Oxidation (FAO) Controls Endothelial Cell (EC) Permeability. *Mol Cell Proteomics*. 2015;14(3):621-634. doi:10.1074/mcp.M114.045575.
4. Nguyen HM, Mejia EM, Chang W, et al. Reduction in cardiolipin decreases mitochondrial spare respiratory capacity and increases glucose transport into and across human brain cerebral microvascular endothelial cells. *J Neurochem*. 2016;139(1):68-80. doi:10.1111/jnc.13753.
5. Anjum F, Joshi K, Grinkina N, Gowda S, Cutaia M, Wadgaonkar R. Role of Sphingomyelin Synthesis in Pulmonary Endothelial Cell Cytoskeletal Activation and Endotoxin-Induced Lung Injury. *Am J Respir Cell Mol Biol*. 2012;47(1):94-103. doi:10.1165/rcmb.2010-0458OC.
6. Wang Y-F, Hsu Y-J, Wu H-F, et al. Endothelium-Derived 5-Methoxytryptophan Is a Circulating Anti-Inflammatory Molecule That Blocks Systemic InflammationNovelty and Significance. *Circ Res*. 2016;119(2):222-236. doi:10.1161/CIRCRESAHA.116.308559.
7. Wang Y, Liu H, McKenzie G, et al. Kynurenine is an endothelium-derived relaxing factor produced during inflammation. *Nat Med*. 2010;16(3):279-285. doi:10.1038/nm.2092.
8. Venkataraman K, Lee Y-M, Michaud J, et al. Vascular Endothelium As a Contributor of Plasma Sphingosine 1-Phosphate. *Circ Res*. 2008;102(6).

9. Sen S, Roy S, Bandyopadhyay G, et al. γ-Aminobutyric Acid Is Synthesized and Released by the EndotheliumNovelty and Significance. *Circ Res*. 2016;119(5):621-634. doi:10.1161/CIRCRESAHA.116.308645.

10. Urquhart P, Parkin SM, Nicolaou A. Profile of eicosanoids produced by human saphenous vein endothelial cells and the effect of dietary fatty acids. *Prostaglandins, Leukot Essent Fat Acids*. 2001;65(1):15-22. doi:10.1054/plef.2001.0282.

11. Pasaoglu OT, Turkozkan N, Ark M, Polat B, Agilli M, Yaman H. The Effect of Taurine on the Relationship Between NO, ADMA and Homocysteine in Endotoxin-Mediated Inflammation in HUVEC Cultures. *Inflammation*. 2014;37(5):1439-1443. doi:10.1007/s10753-014-9868-3.

12. Schoors S, Bruning U, Missiaen R, et al. Fatty acid carbon is essential for dNTP synthesis in endothelial cells. *Nature*. 2015;520(7546):192-197. doi:10.1038/nature14362.
